# Historic samples reveal loss of wild genotype through domestic chicken introgression during the Anthropocene

**DOI:** 10.1371/journal.pgen.1010551

**Published:** 2023-01-19

**Authors:** Meng Yue Wu, Giovanni Forcina, Gabriel Weijie Low, Keren R. Sadanandan, Chyi Yin Gwee, Hein van Grouw, Shaoyuan Wu, Scott V. Edwards, Maude W. Baldwin, Frank E. Rheindt

**Affiliations:** 1 Department of Biological Sciences, National University of Singapore, Singapore, Singapore; 2 Evolution of Sensory Systems Research Group, Max Planck Institute for Ornithology, Seewiesen, Germany; 3 Bird Group, Department of Life Sciences, Natural History Museum, Tring, United Kingdom; 4 Department of Biochemistry and Molecular Biology, Collaborative Innovation Center of Tianjin for Medical Epigenetics, Tianjin Key Laboratory of Medical Epigenetics, School of Basic Medical Sciences, Tianjin Medical University, Tianjin, Chin; 5 Jiangsu Key Laboratory of Phylogenomics and Comparative Genomics, School of Life Sciences, Jiangsu Normal University, Xuzhou, China; 6 Department of Organismic and Evolutionary Biology, Museum of Comparative Zoology, Harvard University, Cambridge, Massachusetts, United States of America; Wageningen University & Research, NETHERLANDS

## Abstract

Human activities have precipitated a rise in the levels of introgressive gene flow among animals. The investigation of conspecific populations at different time points may shed light on the magnitude of human-mediated introgression. We used the red junglefowl *Gallus gallus*, the wild ancestral form of the chicken, as our study system. As wild junglefowl and domestic chickens readily admix, conservationists fear that domestic introgression into junglefowl may compromise their wild genotype. By contrasting the whole genomes of 51 chickens with 63 junglefowl from across their natural range, we found evidence of a loss of the wild genotype across the Anthropocene. When comparing against the genomes of junglefowl from approximately a century ago using rigorous ancient-DNA protocols, we discovered that levels of domestic introgression are not equal among and within modern wild populations, with the percentage of domestic ancestry around 20–50%. We identified a number of domestication markers in which chickens are deeply differentiated from historic junglefowl regardless of breed and/or geographic provenance, with eight genes under selection. The latter are involved in pathways dealing with development, reproduction and vision. The wild genotype is an allelic reservoir that holds most of the genetic diversity of *G*. *gallus*, a species which is immensely important to human society. Our study provides fundamental genomic infrastructure to assist in efforts to prevent a further loss of the wild genotype through introgression of domestic alleles.

## Author summary

The red junglefowl *Gallus gallus* from tropical Asia is the ancestral form of the chicken, which is arguably the most important domestic animal on Earth. Here we discovered an increase in genetic exchange between red junglefowl and chickens in recent years, making wild populations increasingly domestic genomically. By using whole genome sequences of chickens and red junglefowl from two time points, we show that domestic introgression has increased across the wild range at varying levels over the course of a century. We also identified genes that might have played a role in the domestication of the species using the samples from a century ago, in which domestic contribution was lower than in modern day samples. We found eight genes under selection which are involved in development, reproduction and vision, and which might be fundamental to the nature of domestic chickens as distinct from their ancestral wild counterparts. Our study brings to light the current and ongoing loss of the wild junglefowl genotype, suggesting that efforts may be needed to safeguard its full genetic diversity.

## Introduction

Human activities over the last few hundred years have exerted an unprecedented impact on the environment and biodiversity [1–6]. Among the least known repercussions of the modern environmental crisis is a marked increase in anthropogenically mediated genetic introgression between animals [7–9]. As urbanization and habitat loss accelerate [7,10], biotic distribution ranges contract or expand, placing species that had previously been isolated by natural barriers into contact, and leading to a rise in introgressive gene flow [9,11]. The documentation of this increase in genetic admixture remains in its infancy as the investigation of historic DNA samples has posed numerous challenges [12,13]. A successful comparison of genomes at different time points may provide insights into the long-term effects of human-induced introgression.

Red junglefowl (*Gallus gallus*) are the wild, ancestral form of the chicken. The origin of their domestication has been placed in Indochina and/or southernmost China [14] within the range of the junglefowl subspecies *spadiceus*, but little is known about the spread of domestication and subsequent genetic exchange between wild junglefowl and domestic chickens. While the discovery of domestication-specific alleles suggests continual and fairly strong reproductive isolation between chickens and wild junglefowl [14,15], signatures of genomic admixture indicate that the domestic-wild divide has not always been absolute: several studies have established gene flow from domestic chickens into wild junglefowl, although the opposite scenario remains less well documented [16–21]. Although domestic introgression can be beneficial to populations of a wild ancestral species [22,23], preserving wild genotypes of important human domesticates is generally regarded as crucial to preclude sweeps of genomic homogenization and/or a loss of the full range of wild-type genetic diversity [24,25]. Variation within the ancestral population is capable to confer advantages to the domestic population [26]. As such, a loss of wild-type genetic diversity in junglefowl may hinder the safeguarding of one of humanity’s most important food sources. Yet the ongoing global environmental crisis may have promoted an increase of domestic introgression into junglefowl [19], with suggestions that truly unadmixed wild red junglefowl may not have survived when we entered the Anthropocene during the start of the industrial revolution [27].

**Fig 1:**
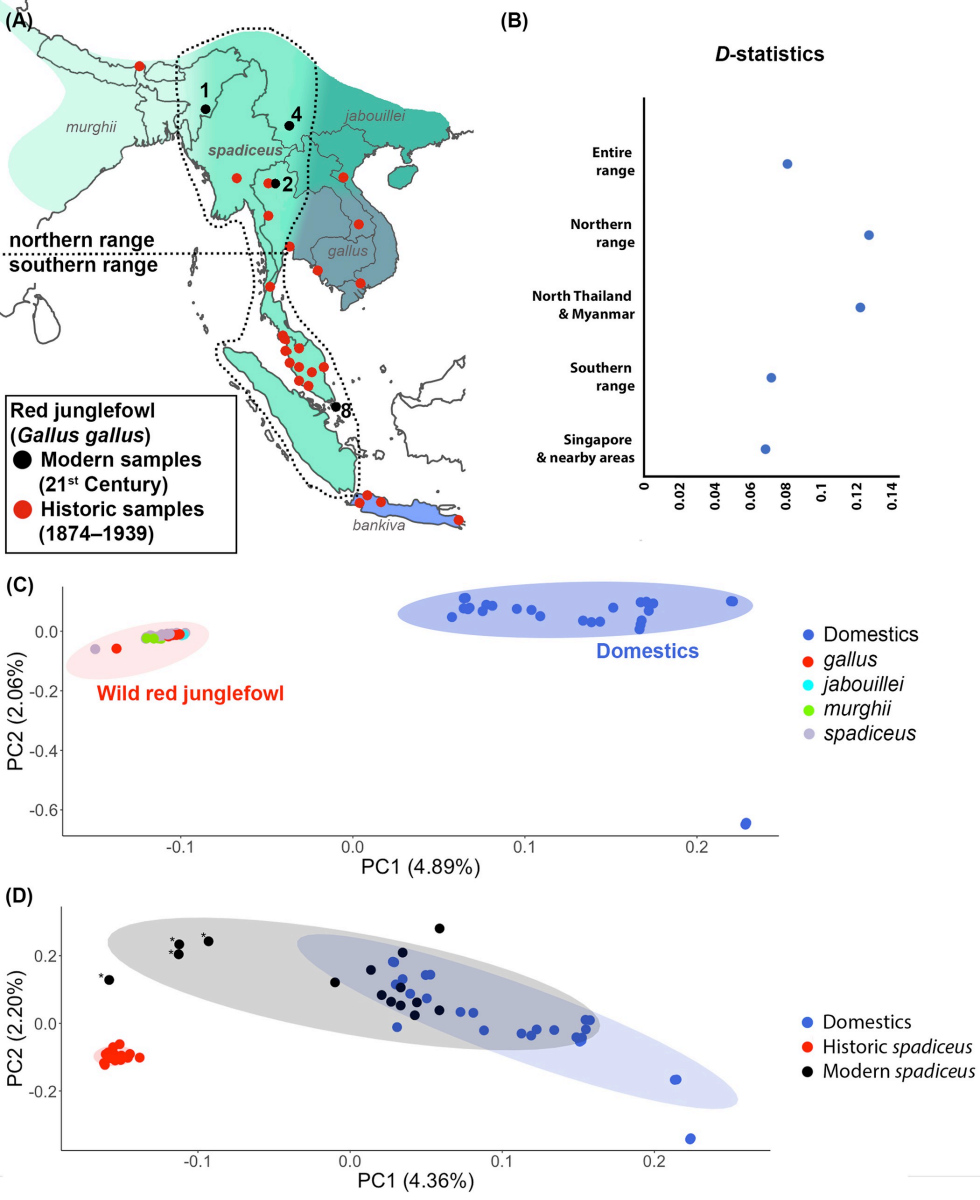
Geographic provenance of samples and overall genetic profile illustrating intensification of domestic contribution into wild red junglefowl. (A) Distribution map of all five red junglefowl (*Gallus gallus*) subspecies following McGowan et al. (2020) [83]. Circles refer to collection localities for the samples used in this study. The numbers next to the black circles are the sample size for each modern red junglefowl population (sample size was 1 for all red circles). The distribution of subspecies *spadiceus*, the putative ancestor of modern chickens, is encircled by a stippled line, and divided into its northern and southern range as defined in this work. (B) *D-*statistics testing for excess allele sharing between domestic chickens and red junglefowl of the ancestral subspecies *spadiceus* using the phylogeny (((*spadiceus*-historic, *spadiceus*-modern), chicken), *Bambusicola thoracicus*) from various parts of the range of *spadiceus* (from top to bottom): the ‘entire range’ of *spadiceus* (n = 85), ‘northern range’ (see map in panel *a*) (n = 61), northern Thailand and Myanmar only (n = 56), ‘southern range’ (see map in panel *a*) (n = 75), and Singapore plus adjacent Malay Peninsula (n = 60). All D-values are significantly non-zero (|Z|>3), indicating a strong signal of domestic introgression into modern samples. (C) Principal component analysis (PCA) of historic samples of red junglefowl and modern domestic chickens (n = 61) based on 7,492,873 SNPs. Javan subspecies *bankiva* removed from plot because it emerged in widely-divergent position (S2 Fig). (D) PCA of subspecies *spadiceus* and domestic chickens (n = 62) based on 2,709,190 SNPs. Individuals labelled with an asterisk (*) pertain to four modern Singaporean junglefowl inferred to have little domestic ancestry (see Fig 2). The percentage of total variation explained by each principal component is shown in brackets. See methods for explanation on each dataset’s sample size. The map was obtained from the R package *rnaturalearth* using Natural Earth medium scale data (1:50m) from https://www.naturalearthdata.com/downloads/.

To investigate the extent of domestic introgression in modern red junglefowl populations, we compared the genomes of 51 chickens with 63 junglefowl–most of the latter dating back >100 years. We ensured a representation of all five extant subspecies of wild *G*. *gallus* in our sampling, and maximized geographic coverage from across the entire historic distribution of the species (Fig 1A and S1 Table). All in all, there are three main groups being compared in this study–(1) historic red junglefowl from a century ago (S1 Table), (2) modern red junglefowl collected at the beginning of this century, and (3) domestic chickens collected in the same time period as the previous. By using genomes of red junglefowl from a century ago as reference points for the wild genotype, hailing from a time when there was a larger area of wild habitats and fewer chickens to interbred with, we quantified excess domestic introgression in modern wild populations. We then identified multiple domestication markers in which chickens are deeply differentiated from wild junglefowl, regardless of breed or geographic provenance, with eight genes under selection. The latter are involved in pathways dealing with the nervous system, reproduction and vision. Our study sheds light on the impact of human-influenced introgression on wild junglefowl and provides fundamental genomic infrastructure to assist future efforts to maintain a substantial pool of their wild genotype.

## Results

We sequenced the genomes of 45 historic red junglefowl, with a total of four historic samples (1 *bankiva*, 1 *murghi*, 2 *spadiceus*) dropping out due to high missingness or severe *post mortem* damage. The final quality filtered reads were around 89 bp long on average, with an average genomic coverage of 7.5X.

### Deepest genomic division is between wild red junglefowl and chickens

Various analytical approaches indicated that the deepest population-genomic divergence in *G*. *gallus* is between chickens and their wild counterparts (Figs 1C and S1) [19,28]. Within our historic wild *G*. *gallus* samples, the subspecies *bankiva* forms a distinct cluster from the other subspecies (S1 and S2 Figs). For the remaining taxa, there seems to be an overall clinal geographic structure from Indian *murghi* to Southeast Asian *spadiceus*, with the latter being closer to domestic chickens (Figs 1C and S2). This result emerged irrespective of the inclusion of chickens from extremely different geographic backgrounds and pedigrees (S1 Fig and S2 Table). The phylogenomic tree constructed from >1 million genome-wide markers found all domestic individuals forming one well-supported clade within the *G*. *gallus* complex (S1 Fig). Using various approaches, we did not find any historic artifact biasing our data (S2 and S3 Figs).

### Historic DNA reveals higher domestic introgression in modern wild junglefowl versus pre-Anthropocene samples

Principal component analysis (PCA) of almost 3 million genome-wide markers suggests that present-day wild populations possess more domestic alleles compared to populations from a century ago (Fig 1D). Here, we limited our analysis to only *G*. *g*. *spadiceus*, the putative ancestral subspecies domestic chickens originated from [14], to reduce artifacts of population structure from other subspecies (S2 Fig). Observed heterozygosity (*H*) has decreased drastically and Tajima’s D values have become positive from historic (*H* = 0.0470, Tajima’s D = -1.15996) to modern *G*. *g*. *spadiceus* (*H* = 0.00464, Tajima’s D = 0.470075), reflecting a loss of allelic diversity in modern *G*. *g*. *spadiceus*. The corresponding statistics for our panel of domestic chickens were mostly similar to modern *G*. *g*. *spadiceus* (*H* = 0.00503, Tajima’s D = -9.32).

Within *G*. *g*. *spadiceus*, modern samples seem to exhibit a clinal population structure from the south (Singapore) to the north of the subspecies range (northeastern India), with seemingly increasing domestic contributions (Figs 1D and S2). The purest examples of modern free-roaming junglefowl exhibit a genomic profile that is almost fully embedded with the historic wild population, while other modern free-roaming samples from throughout the range of *spadiceus* have profiles that cluster much more closely with domestics (Fig 1D), indicating substantial heterogeneity in the level of introgression, sometimes even within modern wild populations.

We quantified levels of excess domestic introgression into modern *spadiceus* populations by computing *D-*statistics using the topology (((*spadiceus*-historic, *spadiceus*-modern), chicken), Chinese bamboo partridge *Bambusicola thoracicus*) [29]. We carried out multiple *D-*statistics calculations using different subsets of populations across the range of *spadiceus* to ensure that population structure did not affect our tests. While these analyses are unable to evaluate the level of domestic introgression that was already present in pre-Anthropocene wild red junglefowl from one century ago, we showed that modern populations of the subspecies *spadiceus* exhibit a significant excess of such introgression as compared to populations from just before the Anthropocene (Fig 1B and S3 Table).

**Fig 2:**
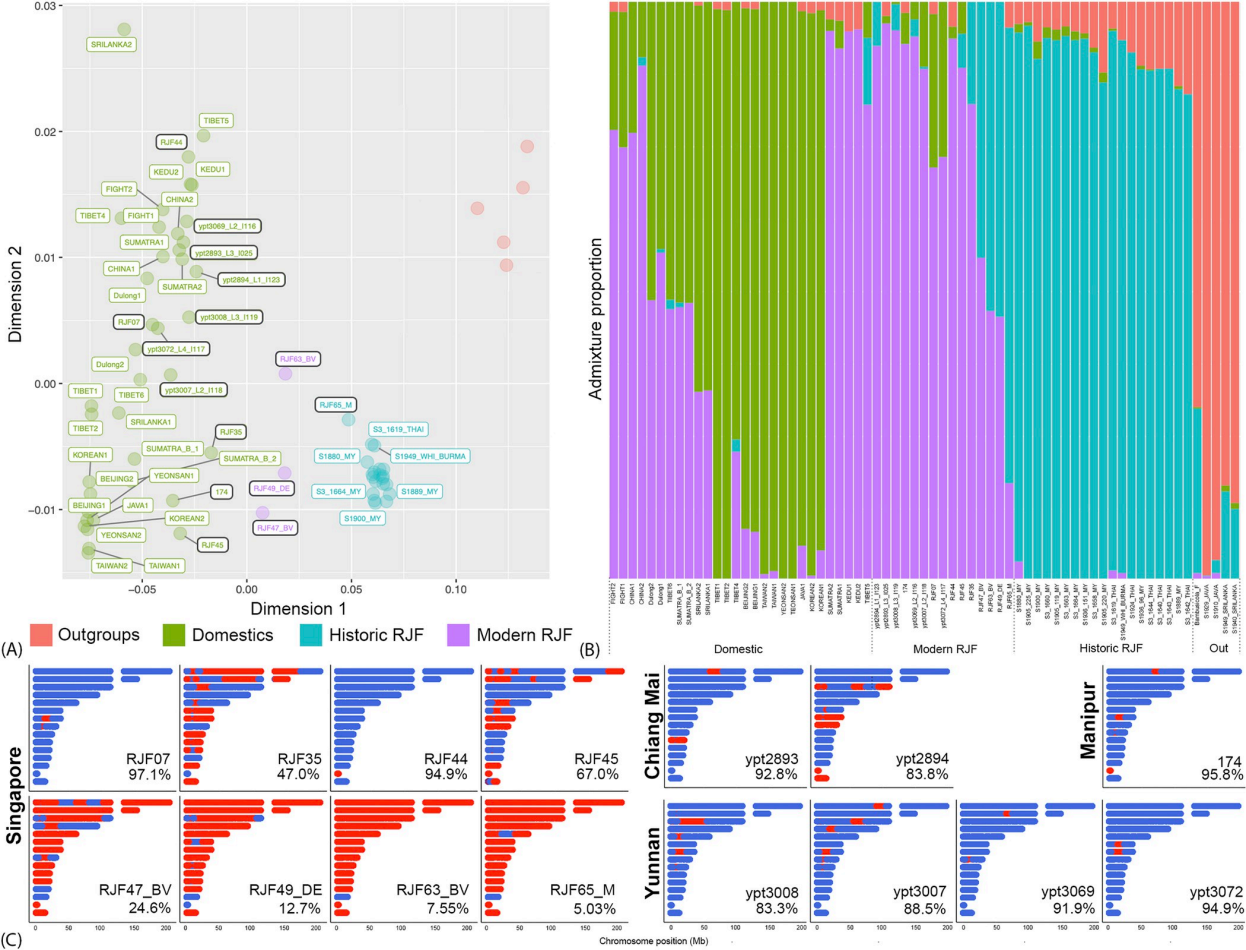
Ancestry inference of 15 representative modern red junglefowl from four localities across the range of *Gallus gallus spadiceus*. (A) Multi-Dimensional Scaling plot from Struct-f4 (*K* = 4) assuming no admixture based on 2,603,726 SNPs. Modern red junglefowl samples are highlighted with a black frame; historic red junglefowl samples are to the right of the modern ones (teal), and domestic chicken samples to the left (green). The color legend indicates which population each sample is assigned to regardless of actual identity (RJF = red junglefowl). (B) Individual ancestry profiles from Struct-f4 (*K* = 4) based on 2,603,726 SNPs. Colors refer to genomic contributions as inferred in 2A. ‘Out’ refers to the outgroups used in the analysis. (C) Local ancestry inference based on 143,526 SNPs using Efficient inference of local ancestry (EILA). Only the top 15 chromosomes (Chr 1, 2, 3, 4, 5, 6, 7, 8, 9, 10, 12, 13, 14, 16, 20) with the most SNPs called are illustrated. Blue coloration refers to domestic ancestry and red coloration refers to wild ancestry. The percentage of genome-wide SNPs with domestic ancestry is given on the bottom right.

When inferring domestic versus wild ancestry across around 2.7 million SNPs using the program Struct-f4 [30], a pattern emerged in which a substantial proportion of modern individuals from throughout the range of *spadiceus* were characterised by a large amount of domestic introgression, reflecting our PCA results (Figs 1D and 2A). Global ancestry inference using the same program found most modern red junglefowl to be distinct from the other populations, with contributions from domestic chickens (20–50%) and historic red junglefowl (45–80% in the four genomically most “wild-type” junglefowl individuals from Singapore) (Fig 2B). Local ancestry inference with almost 150,000 single nucleotide polymorphisms (SNPs) using the program EILA (Efficient inference of local ancestry) [31] found the Singaporean population broadly heterogeneous, with percentages of domestic-introgressed alleles ranging from 5–97% depending on individual (Fig 2C). All individuals screened from northern Thailand (Chiang Mai), northeast India (Manipur) and southwest China (Yunnan) displayed percentages of domestic-introgressed alleles between 83%–96% (Fig 2C). When restricting EILA analysis to using only commercial farm breeds as the domestic reference, a similar trend emerged, but the percentages of domestic contribution fell to 30–70% (S4 Table and S4 Fig). While the accuracy of estimating local ancestry sources in the genome remain contentious, the trend obtained from our EILA analysis is reflective of our global ancestry estimation.

### Identification of possible domestication loci under selection in chickens

**Fig 3:**
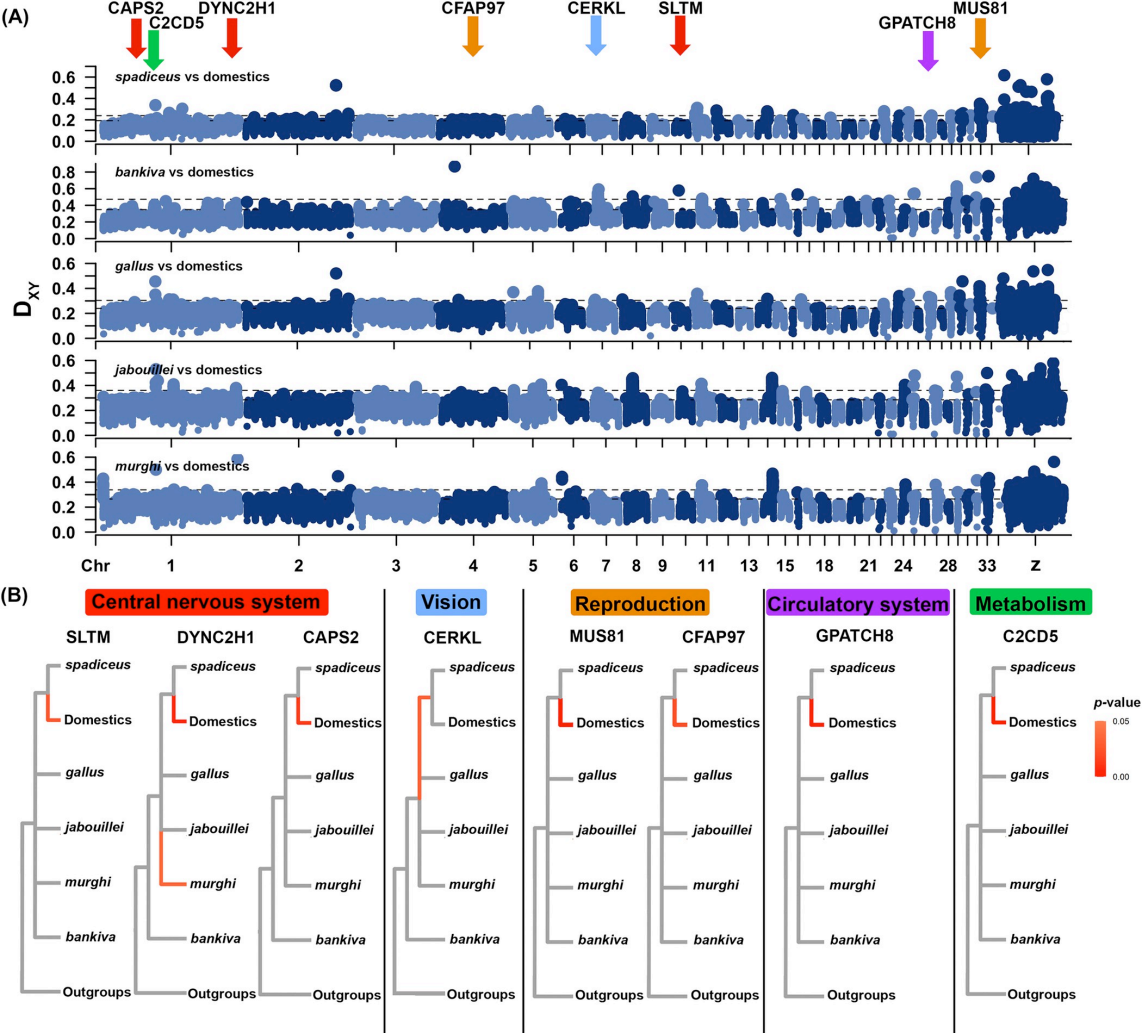
Positive selection on genomic islands of differentiation between chickens and wild red junglefowl. (A) D_XY_ (pairwise divergence) between historic wild individuals of each red junglefowl subspecies versus modern chickens in 50kb sliding windows with a step size of 10kb across the chicken reference genome. Alternating hues of blue denote different chromosomes and the horizontal black dotted lines denote the 99^th^ percentile of D_XY_ of autosomes (bottom) and the Z chromosome (top). Arrows indicate genic regions located within genomic islands of high differentiation between chickens and junglefowl which are under selection in domestic chickens; their colors refer to the main function of genes following (B). (B) Branches under positive selection that were inclusive of domestic chickens calculated using aBSREL (red: uncorrected *p-*value ≤ 0.05) for genes that are highly divergent between chickens and wild junglefowl. Seven outgroups across the avian phylogeny were used in this analysis (signatures of selection in outgroups not depicted, see S6 Fig).

Following from our evidence that domestic introgression into wild red junglefowl has increased in modern times, we sought to find genomic areas of elevated divergence between chickens and historic junglefowl. We performed scans of genomic divergence across the chicken reference genome (RefSeq Assembly Accession: GCF_000002315.6) in 50kb sliding windows using D_XY_ as a measure of differentiation, comparing domestics to wild historic samples of each of the five red junglefowl subspecies (Fig 3). We also used F_ST_ as another measure of differentiation, but preliminary plot inspection indicated an excess of potential domestication loci which may be an artifact of the difference in sample size among populations (S5 Fig). We chose a wide range of domestics from farm breeds to village chickens to reduce signals associated with post-domestication breed formation and adaptive differentiation. Most genomic divergence between domestics and wild individuals was at a roughly constant background level, occasionally disrupted by so-called ‘islands of extreme genomic divergence’ [32,33]. We harvested regions with D_XY_ sliding window values that exceed the 99^th^ percentile for each pairwise comparison and in this way identified 883 potential domestication-related genic regions.

These loci were tested for positive selection using aBSREL with seven outgroups across the avian phylogeny [34]. A total of 112 genes were found to be under selection in the *Gallus gallus* complex, with a subset of eight solely in domesticated chickens not including selection signals in the outgroups (Figs 3 and S6 and Tables 1 and S5). Genes identified as deeply divergent and under selection between domestics and historic wild samples of various subspecies play an important role in such processes as vision maintenance (*CERKL* [35]), reproduction (*CFAP97* [36], *MUS81* [37]), metabolism (*C2CD5* [38]), nervous system signaling (*CAPS2* [39]) and development (*DYNC2H1* [40], *SLTM* [41], *GPATCH8* [42]) (Fig 3 and Table 1). With the exception of *CERKL*, which emerged as under selection in both wild *spadiceus* and chickens, the other genes we identified are only under selection in domestic chickens (Table 1). These eight genes also showcase positive selection in the outgroups (S6 Fig).

**Table 1:** Function of genes located on genomic islands of high differentiation between chickens and junglefowl which are under selection in chickens. Only CERKL was found to be under selection in both chickens and wild *spadiceus*; the remainder were found to be under selection uniquely in chickens. Values are rounded to 3 significant figures (s.f.).

Gene	Phenotype/Effect	Node/taxon under selection	Uncorrected *p-*value (3 s.f.)	Corrected *p-*value (3 s.f.)
*CERKL*	Reducing retinal degeneration as a result of oxidative damage [35]	(*G*. *g*. *spadiceus*, chicken)	0.0322	-
*DYNC2H1*	Formation of cilia, involved in Sonic hedgehog pathway [40]	chicken	0.00626	-
*SLTM*	Regulates GLI factor in Sonic hedgehog pathway [41]	chicken	0.0278	-
*C2CD5*	Involved in glucose uptake by cells, regulates body weight and appetite control [38]	chicken	0.00	0.00
*CFAP97*	Involved in sperm function and fertility [36]	chicken	0.0223	-
*GPATCH8*	Mutation causes high uric acid in blood and bone [42]	chicken	0.0161	-
*CAPS2*	Modulates synaptic activities via LDCV exocytosis pathway [39]	chicken	0.0143	-
*MUS81*	Involved in DNA repair during meiotic division [37]	chicken	0.00130	0.0117

By comparing the genome position of the eight genes under selection (Table 1) with our local ancestry results (Fig 2C), we deduced the most likely origin of these genes in the 15 modern red junglefowl individuals included. Out of the eight genes, we were unable to infer the local ancestry for *MUS81* and *GPATCH8* due to a lack of SNPs in the surroundings of their genomic location in EILA. For the other six genes, the majority of all 15 modern red junglefowl screened possessed a copy of domestic ancestry (*SLTM*– 73.3%, *CFAP97*–80%, *CAPS2*–66.7%, *C2CD5*–60%, *DYNC2H1*–66.7%) with only one exception (*CERKL*– 20%), underpinning the veracity of our approach of using historic red junglefowls as a reference. If these modern individuals had been chosen for domestic selection scans, these genes would not have been flagged as potential candidates as they have already been homogenized in modern junglefowl via domestic introgression.

## Discussion

### Increasing levels of domestic introgression threaten the modern wild genotype of *G*. *gallus*

The deep genetic rift between domestics and all wild junglefowl may appear unusual because the different subspecies of red junglefowl have had hundreds of thousands of years to differentiate, whereas domestication is only thought to date back approximately 4,000 to 10,000 years [27,43,44] (Figs 1 and S1). This rift is indicative of pronounced long-term gene flow among all adjoining continental subspecies of the red junglefowl into historic times, preventing the formation of any deep population-genomic barriers between wild populations. Furthermore, this domestic-wild rift is likely an effect of a strong bottleneck during domestication similar to other domestic animals [45,46]. This clear distinction between our historic *G*. *gallus* individuals and modern chickens suggests that there may have been some form of spatial isolation between chickens and the historic wild junglefowl selected for this study, making the latter an appropriate representative of the wild genotype in further comparisons.

The substantial increase in domestic introgression over the span of only a few decades does not bode well for safeguarding the wild allelic diversity of *G*. *gallus* [20,47,48]. This intensification of domestic admixture is likely driven by anthropogenic change to the native environment of *G*. *gallus*, which has experienced a high level of habitat loss following the relentless human encroachment into tropical Asia’s last wilderness areas in recent decades [49–51]. While the increase in domestic introgression is heterogenous throughout the range, all modern populations will–by now–be in proximity to released domestic chickens that are free to interbreed with them. Our data suggest that intensifying introgression from highly homogenised domestic stock into the diverse wild stock has led to an overall loss of heterozygosity by an order of magnitude. This result may be counter-intuitive at first, given that outbreeding and introgression are theoretically expected to lead to increasing heterozygosity. However, chickens, despite their great phenotypic diversity and population size, are known to have undergone an extreme bottleneck and are genomically impoverished akin to other domestic animals, as reflected by their low heterozygosity and Tajima D values in our study. It is likely the introduction of highly homogenised domestic alleles into the wild red junglefowl gene pool that has caused wild-type genetic diversity to plummet so precipitously.

As a caveat, our conservative assignment of some samples of questionable provenance to ‘domestic village chickens’ (see methods) may have led to an overestimate of the absolute level of domestic introgression. Estimation of local ancestry using only commercial farm samples reduced the domestic contribution by about 30% in some samples (S4 Table). However, this potential bias is incompatible with the stark contrast between the clear genomic division between historic junglefowl and chickens as compared to the more blurred division when modern junglefowl are added (Fig 1), which is clearly due to excess introgression in modern samples. A previous study has found a correlation between plumage and genomic makeup in a chicken-junglefowl cline [19], and while we have photographic evidence only for some of the modern red junglefowl included in this study, this evidence suggests that the samples that cluster closer to domestics do exhibit some phenotypic differences from the ancestral wild phenotype (S7 Fig).

An increase in domestic introgression would allow the wild populations to continue to persist. Indeed, there are documented cases of adaptive introgression aiding wild ancestral populations of domesticated animals in their survival (reviewed in [52]). At the same time, it is unknown whether such a positive evolutionary development would apply in junglefowl. More localized studies on chicken-junglefowl interactions [19] and studies concentrating on single traits [21] have shown that at least some of the domestic admixture into junglefowl may fairly be characterised as adaptive introgression. However, an extensive phenotypic dataset combined with genomic data would be required to conclusively address this question. In the meantime, the steep reduction in genetic diversity following in the wake of domestic introgression (see above) raises concerns that the admixture process may lead to a dangerous reduction of allelic diversity in the wild type.

### The loss of domestication markers due to domestic introgression

The quest for domestication markers is important from two perspectives: (1) it highlights genes and other genomic motifs which render chickens what they are, and which help us define the most important traits underlying domestication; (2) it assists in future attempts to diagnose wild populations which have experienced little admixture with domestic alleles and retained some of the most important genomic architecture characterizing the wild genotype. The search for these loci has been a hot biological topic for decades [14,15,25] and has routinely involved comparisons between modern wild and domestic populations [14,15,25]. While some domestication markers selected for in domestics may be selected against in wild population, allelic homogenization following extensive domestic introgression into modern wild populations could mask many other loci that have played an important role in the original domestication process. The high proportion of modern red junglefowl possessing the domestic copy of genes under selection in domestic chickens is consistent with such a scenario (Table 1 and Fig 2). While we do not possess ancient genomes of red junglefowl, genes identified using historic *G*. *gallus* samples as opposed to modern ones would suffer this problem to a much lower extent due to their less pronounced levels of introgression. As such, the analysis of historic *G*. *gallus* samples is crucial to shedding light on the differences between the domestic chicken and its wild counterpart.

### Genes involved in the nervous system are dissimilar between red junglefowl and chickens

Our study identifies four genes involved in the nervous system that are selected for in chickens but not their wild counterpart. Both *DYNC2H1* and *SLTM* code for proteins involved in the Sonic hedgehog pathway that are critical in the formation of organs and the central nervous system during development by regulating neural crest cells [53–55]. We also identified two other genes with relevance to the nervous system–*CAPS2* modulates synaptic activities [39] and *C2CD5* regulates body weight and appetite in the hypothalamus [38]. These properties are not surprising given that tameness is one of the main characteristics being selected for during domestication [56,57].

Chickens are known to have poorer vision compared to their wild counterparts [58], with a handful of vision genes under positive selection that have been proposed to cause this phenotype [59]. Here we add another vision gene, *CERKL*, that plays a role in reducing oxidative damage on the retina, which is equally under positive selection [35]. Interestingly, wild *spadiceus* also seem to be experiencing positive selection in this gene, either due to (1) introgression of the domestic copy of *CERKL* into wild *spadiceus*, or (2) because *spadiceus* may naturally be endowed with a weaker vision.

A previous study suggested that *TSHR*, a gene encoding for the thyroid-stimulating hormone receptor protein, could be a domestication marker based on its near fixation in chickens compared to *G*. *g*. *spadiceus* [15], a result contradicted by later research suggesting that this pattern is merely a by-product of increasing allele frequency in wild populations [14]. This latter gene is also not flagged in our analyses.

Using genomes of historic specimens from throughout the range of red junglefowl, we were able to (1) demonstrate an intensification of introgression from chickens into modern red junglefowl across the Anthropocene and (2) identify eight candidate genes under selection in chickens. Given that our historic red junglefowl samples exhibited less domestic admixture compared to modern fowl, we were able to identify possible genes in our selection scan that would remain intractable in comparisons involving only modern junglefowl, as the latter’s genomes are frequently homogenized due to extensive domestic introgression. These candidate genes will be critical for future genomic enquiries to add to our understanding of the functional differences between domestic chickens and wild junglefowl.

## Materials and methods

### Population sampling

We obtained 45 dried toepad samples of *G*. *gallus* collected between 1874 and 1939 across its native range preserved at the Natural History Museum at Tring (UK) and Lee Kong Chian Natural History Museum (Singapore) (Fig 1 and S1 Table). The level of domestic introgression in historic samples is likely lower compared to modern-day samples given that habitat encroachment in their native range was less severe in the past, allowing us to use them as a reference point to quantify excess modern introgression. We also incorporated an additional 69 samples of red junglefowl and various chicken breeds available from GenBank, including eight free-roaming *G*. *g*. *spadiceus* from Singapore, seven farm individuals and two historic samples from peninsular Malaysia from Wu et al. (2020) [19] along with 8 *G*. *g*. *spadiceus* from Wang et al. (2020) [14] (S2 Table), adding them to our dataset of 45 historic *G*. *gallus* for a total of 114 samples. We additionally included sequences of one *Bambusicola thoracicus* individual from GenBank as an outgroup [60] (S2 Table), leading to a total dataset of 115 samples. It is difficult to determine the origin of red junglefowl samples from online databases without phenotypic validation. Therefore, we have opted in favor of a conservative approach whereby samples labelled *G*. *gallus* of uncertain but likely domestic origin were labelled as village chickens (S2 Table) and only kept those samples confidently labelled as modern red junglefowl.

### Data generation and basic processing

#### DNA extraction, library preparation and whole-genome resequencing

Our laboratory protocol was designed to accommodate low-quality DNA extracted from the toepads of historic specimens, and to account for sources of potential DNA contamination. We extracted genomic DNA of toepad material from the 45 historic specimens using the DNEasy Blood & Tissue Kit (Qiagen, Hilden, Germany) along with a negative control. Subsequently, we created paired-end libraries using the NEBNext FFPE DNA Repair Mix (New England Biolabs, Massachusetts, United States) and the NEBNext Ultra DNA Library Prep Kit for Illumina (New England Biolabs) with the extraction negative control and an additional library negative control. Both protocols were performed under sterile conditions in a dedicated ancient DNA facility with modifications following Chattopadhyay et al. [61].

DNA concentration for each extract and library was quantified using a Qubit 2.0 High Sensitivity DNA assay (Invitrogen, Carlsbad, USA) and sequence length of each library was visualized with an AATI Fragment Analyzer (Advanced Analytical Technologies, Ankeny, USA). We did not detect any library peak in the AATI profiles of the negative controls. Once checked, libraries were sequenced at NovogeneAIT Genomics (Singapore) on an Illumina HiSeq 4000 platform to produce 150 bp paired-end reads.

#### Quality filtering

Initial quality assessment of raw reads was carried out using FastQC (Babraham Bioinformatics, USA).

For all 115 individuals, adaptor trimming was carried out using cutadapt v2.3 [62]. Historic DNA from museum samples is prone to exogenous contamination, such as from bacteria and humans [12]. We removed potential contamination after adaptor removal by mapping our reads to three reference genomes–(1) human (GenBank Assembly Accession: GCA_000001405.28), (2) chicken (RefSeq Assembly Accession: GCF_000002315.6), (3) and a compilation of all available bacterial genomes on the RefSeq database. We extracted reads that mapped uniquely to the chicken reference genome in FastQ_Screen v0.13.0 before continuing [63]. These filtered reads were mapped to the chicken reference genome (RefSeq Assembly Accession: GCF_000002315.6) using BWA-MEM [64]. Low quality reads (MAPQ score <20) were filtered with SAMtools v1.9 to ensure unique mapping [65]. Picard v2.20.2 (http://broadinstitute.github.io/picard/) was subsequently used to assign read group information and mark duplicates. The original alignments were then realigned and refined using RealignedTargetCreator and IndelRealigner as implemented in the Genome Analysis Toolkit v3.8–0 (Broad Institute, USA) [66]. Lastly, we rescaled the quality scores of all historic samples using a Bayesian statistical model of DNA damage as implemented in MapDamage 2.0 to account for *post mortem* DNA damage [67]. We found elevated C to T substitutions in the first few bases of read 1 and elevated G to A substitutions in the last few bases of read 2 in the MapDamage report. We proceeded to trimming the first and last five base pairs off each sample’s sequences to ensure the quality of our data before further analysis. The final bam files were checked in Qualimap v2.2.2 for mapping quality and sequencing bias before variant calling [68]. We initially also added 44 modern samples of various subspecies of *G*. *gallus* from Wang et al. (2020) [14] but found them, specifically *murghi* due to much lower coverage, to exhibit a puzzling signal of population structure far outside all our other samples and therefore opted to remove them to preclude artifacts (S2 Fig).

#### Population genomic approaches

We explored the possibility of historic artifacts based on the degraded nature of our samples by projecting the variation of historic samples on the basis of the higher quality modern samples using smartpca in EIGENSOFT (http://www.hsph.harvard.edu/alkes-price/software/) (S2 and S3 Figs). We did not find any historic artifacts biasing our data. Therefore, we then used ANGSD v0.923 (settings: -uniqueOnly 1, -remove_bads 1, -only_proper_pairs 1 -SNP_pval 1e-6, -minMapQ 30 -minQ 30 -minMaf 0.03 -minInd n -minIndDepth 3 -geno_mindepth 3) for SNP capture on different *G*. *gallus* datasets. This software has been specifically designed to conduct population genetic analysis with low coverage genome data, and was hence deemed suitable for the nature of our historic samples [69,70]. For initial data exploration, a concatenated SNP tree was created in RAxML with Lewis correction using the GTRGAMMA model with mostly domestic and historic wild samples, and four modern samples as representatives [71]. We also conducted PCA on the *G*. *gallus* samples to assess population subdivision using PCAngsd [72]. We found the PCA to be polarized by the Javan subspecies *G*. *g*. *bankiva* (n = 5) (S2 Fig). We also found certain samples of commercial breeds and village chickens (n = 22) to exert high polarization on the visualization when added to the dataset: these latter samples, while still within the domestic cluster, scattered widely and illustrated population structure within the cluster. Moreover, we found an additional seven samples to display high missingness at called loci: five historic wild, one modern wild and one domestic sample from GenBank. To achieve a clearer spread across *G*. *gallus* subspecies, we removed these subpar individuals together with the samples of *G*. *g*. *bankiva* (n = 5), modern *G*. *gallus* (n = 15) and the selected commercial breeds (n = 22) prior to conducting a PCA with only historic *G*. *gallus* with the same filters (n = 61; 7,492,873 SNPs). We also conducted PCA on only *G*. *g*. *spadiceus* with the following filters: uniqueOnly 1, -remove_bads 1, -only_proper_pairs 1 -SNP_pval 1e-6, -minMapQ 30 -minQ 30 -minMaf 0.03 -minInd 46 -minIndDepth 3 -geno_mindepth 3 (n = 62; 2,709,190 SNPs). We only used a subset of domestic breed individuals for the PCAs to retain valuable analytical PCA space for the red junglefowl samples of interest. For the latter analyses, we included all breeds available to represent domestic chickens (n = 50).

We additionally conducted Bayesian clustering in STRUCTURE with a random subset of 100,000 SNPs, employing the wrapper Structure_threader v1.2.4 to parallelize all runs on the *G*. *gallus* dataset (n = 61 + 4 modern *spadiceus*) [73,74]. Clustering analysis was run for *K* = 1 to 10 with 10 replicates for each *K*, a burn-in of 100,000 generations and 500,000 further Monte-Carlo Markov Chain (MCMC) generations. To aggregate replicates for each *K*, we ran the STRUCTURE output through CLUMPAK using the FullSearch algorithm for *K* = 1 to 4 and Greedy algorithm for *K* = 5 to 10 [75].

We tested for excess allele sharing between *G*. *g*. *spadiceus* and the domestic population by applying pairwise ABBA-BABA tests as implemented in ANGSD with error correction and ancient transition removal [76]. We used *B*. *thoracicus* as an outgroup because other members of the genus *Gallus* may have introgressed with *G*. *gallus* [15,77,78]. The tree topology tested was thus (((RJF1, RJF2), domestics), outgroup), where RJF refers to either modern or historic *G*. *g*. *spadiceus* from various localities.

We inferred local ancestry of 15 modern *G*. *g*. *spadiceus* included in this study using EILA in R [31]. We used historic *G*. *g*. *spadiceus* and domestic chickens as ‘ancestral’ populations of modern *G*. *g*. *spadiceus*. We called 143,526 SNPs with ANGSD (-uniqueOnly 1, -remove_bads 1, -only_proper_pairs 1, minMapQ 30, -minQ 20, -setMinDepth 2, -SNP_pval 1e-6, -skipTriallelic 1, -minInd 15). To calculate the percentage of domestic SNPs, SNPs with domestic ancestry were summed across all major chromosomes over the total number of SNPs called. We also repeated the EILA analyses using only commercial farm breeds as the representation for the domestic ‘ancestral’ population.

We inferred global ancestry using 2,603,726 SNPs from the 15 modern *G*. *g*. *spadiceus* samples using Struct-f4 individually [30]. Struct-f4 considers allele sharing, population-specific drift and potential inbreeding, all of which may distort classical population clustering analyses. We first converted the tped file into multiple 5Mb-long files using Tped2Structf4.pl before running the calc-f4 C script (-n 67). The prior output was used to run struct-f4 (-K 4 -m 200,000) in R. All scripts are included in the Struct-f4 package [30].

To identify specific regions showing excess allele sharing between each *G*. *gallus* subspecies and domestic stock, we used D_XY_ as an estimate of differentiation. We first called SNPs that are present in both wild individuals of each *G*. *gallus* subspecies and domestics (-uniqueOnly 1, -remove_bads 1, -only_proper_pairs 1, minMapQ 30, -minQ 20, -setMinDepth 3, -SNP_pval 1e-6, -skipTriallelic 1, -minInd n), with ANGSD using *B*. *thoracicus* as an ancestral reference. We obtained D_XY_ using popgenwindows.py (available at https://github.com/simonhmartin/genomics_general) with a 50 kb sliding window and a step size of 10 kb. We identified D_XY_ values above the 99^th^ percentile of each pairwise comparison, and harvested genes in those regions for all five subspecies for selection tests. Annotated loci or genes present in these selected genomic regions were identified using the chicken reference genome (RefSeq Assembly Accession: GCF_000002315.6). We calculated heterozygosity and Tajima’s D using ANGSD -dosaf 1 and realSFS. Tajima’s D values were later summarized using thetaStat.

Outgroup species for selection tests were the emu *Dromaius novaehollandiae*, tufted duck *Aythya fuligula*, helmeted guineafowl *Numida meleagris*, swan goose *Anser cygnoides*, common pheasant *Phasianus colchicus*, turkey *Meleagris gallopavo*, Japanese quail *Coturnix japonica*, mallard *Anas platyrhynchos* and zebra finch *Taeniopygia guttata*. All sequences were processed using Orthofinder v2.5.2 to remove genes with paralogs [79]. The sequences were realigned and low similarity segments removed using MACSE and HmmCleaner, respectively [80–82]. We instituted an internal gap penalty and missing data penalty, where sequences with large internal gaps and more than 50% missing data were removed using custom scripts. Sequences were then tested for positive selection using aBSREL in HyPhy using the input gene tree topology of ((((((((((*murghi*, *jabouillei*, *gallus*, (domestics, *spadiceus*)), *bankiva*), common pheasant), Japanese quail), turkey), helmeted guineafowl), (swan goose, (tufted duck, mallard))), zebra finch), emu)) [34]. Gene trees may not contain all 14 groups after quality filtering, so we ran aBSREL on all genes that retained at least one outgroup. For visualisation, we mapped selection signals on the input topology to aid in comparsion between all genes. Disregarding positive selection on the outgroups, all genes that exhibit signatures of positive selection on any of the *Gallus* nodes or branches were identified with the primary focus placed on genes that exhibit selection on domestic chickens for further evaluation (S5 Table).

## Supporting information

S1 Fig Population subdivision within *Gallus gallus*. Phylogenetic relationships within *Gallus gallus* obtained from the concatenation of a subset of 1,172,919 single nucleotide polymorphisms harvested from whole genome resequenced samples using maximum-likelihood in RAxML (left). *Bambusicola thoracicus* was used as an outgroup (not shown). Numbers at nodes are bootstrap values. STRUCTURE plots for *K* = 2 to 10 based on 100,000 single nucleotide polymorphisms (right), run for a selection of domestic chickens and for all wild junglefowl samples except the divergent subspecies *bankiva*. For *K* = 2, the orange colour represents the domestic population contribution while blue represents the ‘wild-type’ contribution. Historic samples were collected between 1874 and 1939 and modern samples at the beginning of this century.
(TIF)

S2 Fig Principal component analysis of *Gallus gallus*. The percentage variation explained by each principal component (PC) is shown in brackets. (A) Historic wild junglefowl of all five subspecies. Historic red junglefowl of the Javan subspecies *bankiva* (labeled as ‘Indonesia’) were found to be widely divergent (right-hand side of plot) based on PC1. (B) Historic and modern samples of wild junglefowl of all non-*bankiva* subspecies. Modern and historic samples from many populations of red junglefowl (especially *murghi* from India) show substantial genetic separation. (C) Using the same dataset as in (B), the placement of historic samples was projected using the variation from the modern samples using smartpca. This analytical approach led to a correction of the position of low coverage modern Indian samples of subspecies *murghi*. Meanwhile, the placement of historic samples is similar to (B). Historic samples were collected between 1874 and 1939 and modern samples at the beginning of this century.
(TIF)

S3 Fig Principal component analysis of *Gallus gallus spadiceus* and domestic chicken using smartpca. Placement of historic samples was projected using the variation from the modern samples.
(TIF)

S4 Fig Local ancestry inference based on 422,985 SNPs using Efficient inference of local ancestry (EILA). Only the top 15 chromosomes (Chr 1, 2, 3, 4, 5, 6, 7, 8, 9, 10, 12, 13, 14, 16, 20) with the most SNPs called are illustrated. Blue coloration refers to domestic ancestry, red coloration refers to wild ancestry, purple coloration refers to admixed ancestry.
(TIF)

S5 Fig F_ST_ pairwise divergences between historic wild individuals of each red junglefowl subspecies and modern chickens in 50kb sliding windows with a step size of 10kb across the chicken reference genome. Alternating hues of blue denote different chromosomes and the horizontal black dotted lines denote the 99^th^ percentile of F_ST_ of autosomes (bottom) and the Z chromosome (top)
(TIF)

S6 Fig Patterns of positive selection in domestic chickens calculated using aBSREL (red: uncorrected p-value ≤ 0.05) for genes that are highly divergent between chickens and wild junglefowl. The input tree topology was given as the input gene tree topology of ((((((((((*murghi*, *jabouille*, *gallus*, (domestics, *spadiceus*)), *bankiva*), common pheasant), Japanese quail), turkey), helmeted guineafowl), (swan goose, (tufted duck, mallard))), zebra finch), emu)). Only two outgroups were present after quality filtering in MUS81.
(TIF)

S7 Fig Photographs of the four red junglefowl individuals that cluster closely with the domestic chickens. These individuals do not exhibit the wild phenotype for red junglefowl (see 1st table in Wu et al. 2020 [19]). [Photo courtesy of Gabriel Weijie Low].
(TIF)

S1 Table Sample information of historic museum specimens. (XLSX)

S2 Table List of whole genome resequenced datasets available on Sequence Read Archive, European Nucleotide Archive or chickenSD for *Gallus gallus* and used in this study. Local chickens that do not confer to a breed are labelled as “village”.
(XLSX)

S3 Table Results of introgression tests from chickens into modern *Gallus gallus spadiceus*, relative to historic *G*. *g*. *spadiceus*. (XLSX)

S4 Table Local ancestry inference using Efficient inference of local ancestry (EILA). Comparison of two datasets–(1) Using all domestic samples based on 143,526 SNPs; and (2) using only commercial farm breeds based on 422,985 SNPs. Only the top 15 chromosomes (Chr 1, 2, 3, 4, 5, 6, 7, 8, 9, 10, 12, 13, 14, 16, 20) were included in the calculations.
(XLSX)

S5 Table List of branch/taxa found to be under selection by aBSREL in the genus *Gallus*. (XLSX)

## References

[1] Johnson CN, Balmford A, Brook BW, Buettel JC, Galetti M, Guangchun L, et al. Biodiversity losses and conservation responses in the Anthropocene. Science. 2017;356(6335):270–275. doi: 10.1126/science.aam9317 28428393

[2] Hoegh-Guldberg O, Bruno JF. The impact of climate change on the world’s marine ecosystems. Science. 2010;328(5985):1523–1528. doi: 10.1126/science.1189930 20558709

[3] Brown SC, Wigley TM, Otto-Bliesner BL, Rahbek C, Fordham DA. Persistent Quaternary climate refugia are hospices for biodiversity in the Anthropocene. Nature Climate Change. 2020;10(3):244–248.

[4] Daru BH, Davies TJ, Willis CG, Meineke EK, Ronk A, Zobel M, et al. Widespread homogenization of plant communities in the Anthropocene. Nature Communications. 2021;12(1): 6983. doi: 10.1038/s41467-021-27186-8 34873159 PMC8648934

[5] Emer C, Galetti M, Pizo MA, Jordano P, Verdú M. Defaunation precipitates the extinction of evolutionarily distinct interactions in the Anthropocene. Science Advances. 2019;5(6):eaav6699. doi: 10.1126/sciadv.aav6699 31223648 PMC6584213

[6] Kelly LT, Giljohann KM, Duane A, Aquilué N, Archibald S, Batllori E, et al. Fire and biodiversity in the Anthropocene. Science. 2020;370(6519):eabb0355. doi: 10.1126/science.abb0355 33214246

[7] Lewis SL, Maslin MA. Defining the anthropocene. Nature. 2015;519(7542):171–180. doi: 10.1038/nature14258 25762280

[8] Oliveira R, Randi E, Mattucci F, Kurushima JD, Lyfhyons LA, Alves PC. Toward a genome-wide approach for detecting hybrids: informative SNPs to detect introgression between domestic cats and European wildcats (*Felis silvestris*). Heredity. 2015;115(3):195–205. doi: 10.1038/hdy.2015.25 26103945 PMC4814236

[9] Ottenburghs J. The genic view of hybridization in the Anthropocene. Evolutionary Applications. 2021;14(10): 2342–2360. doi: 10.1111/eva.13223 34745330 PMC8549621

[10] Steffen W, Broadgate W, Deutsch L, Gaffney O, Ludwig C. The trajectory of the Anthropocene: the great acceleration. The Anthropocene Review. 2015;2(1):81–98.

[11] Mank JE, Carlson JE, Brittingham MC. A century of hybridization: decreasing genetic distance between American black ducks and mallards. Conservation Genetics. 2004;5(3):395–403.

[12] Pääbo S, Poinar H, Serre D, Jaenicke-Després V, Hebler J, Rohland N, et al. Genetic analyses from ancient DNA. Annual Review of Genetics. 2004;38:645–679. doi: 10.1146/annurev.genet.37.110801.143214 15568989

[13] Burrell AS, Disotell TR, Bergey CM. The use of museum specimens with high-throughput DNA sequencers. Journal of Human Evolution. 2015;79:35–44. doi: 10.1016/j.jhevol.2014.10.015 25532801 PMC4312722

[14] Wang MS, Thakur M, Peng MS, Jiang Y, Frantz LA, Li M, et al. 863 genomes reveal the origin and domestication of chicken. Cell Research. 2020;30(8):693–701. doi: 10.1038/s41422-020-0349-y 32581344 PMC7395088

[15] Rubin CJ, Zody MC, Eriksson J, Meadows JR, Sherwood E, Webster MT, et al. Whole-genome resequencing reveals loci under selection during chicken domestication. Nature. 2010;464(7288):587–591. doi: 10.1038/nature08832 20220755

[16] Brisbin IL. Concerns for the genetic integrity and conservation status of the Red Jungle Fowl. Tragopan. 1996;4:11–12.

[17] Peterson AT, Brisbin IL. Genetic endangerment of wild Red Junglefowl *Gallus gallus*?. Bird Conservation International. 1998;8(4):387–394.

[18] Gering E, Johnsson M, Willis P, Getty T, Wright D. Mixed ancestry and admixture in Kauai’s feral chickens: invasion of domestic genes into ancient Red Junglefowl reservoirs. Molecular Ecology. 2015;24(9):2112–2124. doi: 10.1111/mec.13096 25655399

[19] Wu MY, Low GW, Forcina G, van Grouw H, Lee BP, Oh RR, et al. Historic and modern genomes unveil a domestic introgression gradient in a wild red junglefowl population. Evolutionary Applications. 2020;13(9):2300–2315. doi: 10.1111/eva.13023 33005225 PMC7513718

[20] Brisbin IL Jr, Peterson AT. Playing chicken with red junglefowl: identifying phenotypic markers of genetic purity in *Gallus gallus*. Animal Conservation. 2007;10(4):429–435.

[21] Eriksson J, Larson G, Gunnarsson U, Bed’Hom B, Tixier-Boichard M, Strömstedt L, et al. Identification of the yellow skin gene reveals a hybrid origin of the domestic chicken. PLoS Genetics. 2008;4(2):e1000010. doi: 10.1371/journal.pgen.1000010 18454198 PMC2265484

[22] Wu DD, Ding XD, Wang S, Wójcik JM, Zhang YI, Tokarska M, et al. Pervasive introgression facilitated domestication and adaptation in the *Bos* species complex. Nature Ecology & Evolution. 2018;2(7):1139–1145. doi: 10.1038/s41559-018-0562-y 29784979

[23] Hinton JW, West KM, Sullivan DJ, Frair JL, Chamberlain MJ. The natural history and ecology of melanism in red wolf and coyote populations of the southeastern United States–evidence for Gloger’s rule. BMC Zoology. 2022;7(1):1–7. 10.1186/s40850-022-00138-5 PMC10127370 37170305

[24] Gering E, Incorvaia D, Henriksen R, Wright D, Getty T. Maladaptation in feral and domesticated animals. Evolutionary Applications. 2019;12(7):1274–1286. doi: 10.1111/eva.12784 31417614 PMC6691326

[25] Qanbari S, Rubin CJ, Maqbool K, Weigend S, Weigend A, Geibel J, et al. Genetics of adaptation in modern chicken. PLoS Genetics. 2019;15(4):e1007989. doi: 10.1371/journal.pgen.1007989 31034467 PMC6508745

[26] Barbato M, Hailer F, Orozco-terWengel P, Kijas J, Mereu P, Cabras P, et al. Genomic signatures of adaptive introgression from European mouflon into domestic sheep. Scientific Reports. 2017;7(1):1–3. 28790322 10.1038/s41598-017-07382-7 PMC5548776

[27] Lawler A. In search of the wild chicken. Science. 2012;338:1020–1024. 23180839 10.1126/science.338.6110.1020

[28] Lawal RA, Al-Atiyat RM, Aljumaah RS, Silva P, Mwacharo JM, Hanotte O. Whole-genome resequencing of red junglefowl and indigenous village chicken reveal new insights on the genome dynamics of the species. Frontiers in Genetics. 2018;9:264. doi: 10.3389/fgene.2018.00264 30079080 PMC6062655

[29] Patterson N, Moorjani P, Luo Y, Mallick S, Rohland N, Zhan Y, et al. Ancient admixture in human history. Genetics. 2012;192(3):1065–1093. doi: 10.1534/genetics.112.145037 22960212 PMC3522152

[30] Librado P, Orlando L. Struct-f4: a Rcpp package for ancestry profile and population structure inference from f4-statistics. Bioinformatics. 2022;38(7):2070–2071. doi: 10.1093/bioinformatics/btac046 35080599 PMC8963280

[31] Yang JJ, Li J, Buu A, Williams LK. Efficient inference of local ancestry. Bioinformatics. 2013;29(21):2750–2756. doi: 10.1093/bioinformatics/btt488 23958727 PMC3799480

[32] Turner TL, Hahn MW, Nuzhdin SV. Genomic islands of speciation in *Anopheles gambiae*. PLoS Biology. 2005;3(9):e285. doi: 10.1371/journal.pbio.0030285 16076241 PMC1182689

[33] Harr B. Genomic islands of differentiation between house mouse subspecies. Genome Research. 2006;16(6):730–737. doi: 10.1101/gr.5045006 16687734 PMC1473184

[34] Smith MD, Wertheim JO, Weaver S, Murrell B, Scheffler K, Kosakovsky Pond SL. Less is more: an adaptive branch-site random effects model for efficient detection of episodic diversifying selection. Molecular Biology and Evolution. 2015;32(5):1342–1353. doi: 10.1093/molbev/msv022 25697341 PMC4408413

[35] Li C, Wang L, Zhang J, Huang M, Wong F, Liu X, et al. CERKL interacts with mitochondrial TRX2 and protects retinal cells from oxidative stress-induced apoptosis. Biochimica et Biophysica Acta (BBA)-Molecular Basis of Disease. 2014;1842(7):1121–1129. doi: 10.1016/j.bbadis.2014.04.009 24735978

[36] Soulavie F, Piepenbrock D, Thomas J, Vieillard J, Duteyrat JL, Cortier E, et al. *hemingway* is required for sperm flagella assembly and ciliary motility in *Drosophila*. Molecular Biology of the Cell. 2014;25(8):1276–1286. doi: 10.1091/mbc.E13-10-0616 24554765 PMC3982993

[37] Ciccia A, McDonald N, West SC. Structural and functional relationships of the XPF/MUS81 family of proteins. Annual Review of Biochemistry. 2008;77:259–287. doi: 10.1146/annurev.biochem.77.070306.102408 18518821

[38] Gavini CK, Cook TM, Rademacher DJ, Mansuy-Aubert V. Hypothalamic C2-domain protein involved in MC4R trafficking and control of energy balance. Metabolism. 2020;102:153990. doi: 10.1016/j.metabol.2019.153990 31666192

[39] Speidel D, Varoqueaux F, Enk C, Nojiri M, Grishanin RN, Martin TF, et al. A family of Ca2+-dependent activator proteins for secretion: comparative analysis of structure, expression, localization, and function. Journal of Biological Chemistry. 2003;278(52):52802–52809. doi: 10.1074/jbc.M304727200 14530279

[40] Toropova K, Zalyte R, Mukhopadhyay AG, Mladenov M, Carter AP, Roberts AJ. Structure of the dynein-2 complex and its assembly with intraflagellar transport trains. Nature Structural & Molecular Biology. 2019;26(9):823–829. doi: 10.1038/s41594-019-0286-y 31451806 PMC6774794

[41] Zhang Z, Zhan X, Kim B, Wu J. A proteomic approach identifies SAFB-like transcription modulator (SLTM) as a bidirectional regulator of GLI family zinc finger transcription factors. Journal of Biological Chemistry. 2019;294(14):5549–5561. doi: 10.1074/jbc.RA118.007018 30782847 PMC6462520

[42] Kaneko H, Kitoh H, Matsuura T, Masuda A, Ito M, Mottes M, et al. Hyperuricemia cosegregating with osteogenesis imperfecta is associated with a mutation in GPATCH8. Human Genetics. 2011;130(5):671–683. doi: 10.1007/s00439-011-1006-9 21594610

[43] Peters J, Lebrasseur O, Deng H, Larson G. Holocene cultural history of Red jungle fowl (*Gallus gallus*) and its domestic descendant in East Asia. Quaternary Science Reviews. 2016;142:102–119.

[44] Xiang H, Gao J, Yu B, Zhou H, Cai D, Zhang Y, et al. Early Holocene chicken domestication in northern China. Proceedings of the National Academy of Sciences. 2014;111(49):17564–17569. doi: 10.1073/pnas.1411882111 25422439 PMC4267363

[45] Vonholdt BM, Pollinger JP, Earl DA, Parker HG, Ostrander EA, Wayne RK. Identification of recent hybridization between gray wolves and domesticated dogs by SNP genotyping. Mammalian Genome. 2013;24(1):80–88. doi: 10.1007/s00335-012-9432-0 23064780

[46] Goedbloed DJ, van Hooft P, Megens HJ, Langenbeck K, Lutz W, Crooijmans RP, et al. Reintroductions and genetic introgression from domestic pigs have shaped the genetic population structure of Northwest European wild boar. BMC Genetics. 2013;14(1):1–0. doi: 10.1186/1471-2156-14-43 23688182 PMC3663677

[47] Lenormand T. Gene flow and the limits to natural selection. Trends in Ecology & Evolution. 2002;17(4):183–189.

[48] Lawson LP, Fessl B, Vargas FH, Farrington HL, Cunninghame HF, Mueller JC, et al. Slow motion extinction: inbreeding, introgression, and loss in the critically endangered mangrove finch (*Camarhynchus heliobates*). Conservation Genetics. 2017;18(1):159–170.

[49] Sodhi NS, Brook BW. Southeast Asian biodiversity in crisis. Cambridge: Cambridge University Press; 2006.

[50] Sodhi NS, Koh LP, Brook BW, Ng PK. Southeast Asian biodiversity: an impending disaster. Trends in Ecology & Evolution. 2004;19(12):654–660. doi: 10.1016/j.tree.2004.09.006 16701328

[51] Sodhi NS, Posa MR, Lee TM, Bickford D, Koh LP, Brook BW. The state and conservation of Southeast Asian biodiversity. Biodiversity and Conservation. 2010;19(2):317–328.

[52] Hedrick PW. Adaptive introgression in animals: examples and comparison to new mutation and standing variation as sources of adaptive variation. Molecular Ecology. 2013;22(18):4606–4618. doi: 10.1111/mec.12415 23906376

[53] Achilleos A, Trainor PA. Neural crest stem cells: discovery, properties and potential for therapy. Cell Research. 2012;22(2):288–304. doi: 10.1038/cr.2012.11 22231630 PMC3271580

[54] Testaz S, Jarov A, Williams KP, Ling LE, Koteliansky VE, Fournier-Thibault C, et al. Sonic hedgehog restricts adhesion and migration of neural crest cells independently of the Patched-Smoothened-Gli signaling pathway. Proceedings of the National Academy of Sciences. 2001;98(22):12521–12526. 10.1073/pnas.221108698 PMC60086 11592978

[55] Sun MR, Chung HM, Matsuk V, Fink DM, Stebbins MJ, Palecek SP, et al. Sonic Hedgehog Signaling in Cranial Neural Crest Cells Regulates Microvascular Morphogenesis in Facial Development. Frontiers in Cell and Developmental Biology. 2020;8:1055. doi: 10.3389/fcell.2020.590539 33117819 PMC7575766

[56] Ahmad HI, Ahmad MJ, Jabbir F, Ahmar S, Ahmad N, Elokil AA, et al. The domestication makeup: Evolution, survival, and challenges. Frontiers in Ecology and Evolution. 2020:103

[57] Dobney K, Larson G. Genetics and animal domestication: new windows on an elusive process. Journal of Zoology. 2006;269(2):261–271.

[58] Roth LS, Lind O. The impact of domestication on the chicken optical apparatus. PLoS One. 2013;8(6):e65509. doi: 10.1371/journal.pone.0065509 23776492 PMC3680433

[59] Wang MS, Zhang RW, Su LY, Li Y, Peng MS, Liu HQ, et al. Positive selection rather than relaxation of functional constraint drives the evolution of vision during chicken domestication. Cell Research. 2016;26(5):556–573. doi: 10.1038/cr.2016.44 27033669 PMC4856766

[60] Tiley GP, Kimball RT, Braun EL, Burleigh JG. Comparison of the Chinese bamboo partridge and red Junglefowl genome sequences highlights the importance of demography in genome evolution. BMC Genomics. 2018;19(1):1–6. 29739321 10.1186/s12864-018-4711-0 PMC5941490

[61] Chattopadhyay B, Garg KM, Mendenhall IH, Rheindt FE. Historic DNA reveals Anthropocene threat to a tropical urban fruit bat. Current Biology. 2019;29(24):R1299–R1300. 31846673 10.1016/j.cub.2019.11.013

[62] Martin M. Cutadapt removes adapter sequences from high-throughput sequencing reads. EMBnet. Journal. 2011;17(1):10–12.

[63] Wingett SW, Andrews S. FastQ Screen: A tool for multi-genome mapping and quality control. F1000Research. 2018;7. doi: 10.12688/f1000research.15931.2 30254741 PMC6124377

[64] Li H. Aligning sequence reads, clone sequences and assembly contigs with BWA-MEM. arXiv:1303.3997v2 [Preprint]. 2013 [cited 2022 April 29]. Available from: 10.48550/arXiv.1303.3997

[65] Li H, Handsaker B, Wysoker A, Fennell T, Ruan J, Homer N, et al. The sequence alignment/map format and SAMtools. Bioinformatics. 2009;25(16):2078–2079. doi: 10.1093/bioinformatics/btp352 19505943 PMC2723002

[66] McKenna A, Hanna M, Banks E, Sivachenko A, Cibulskis K, Kernytsky A, et al. The Genome Analysis Toolkit: a MapReduce framework for analyzing next-generation DNA sequencing data. Genome Research. 2010;20(9):1297–1303. doi: 10.1101/gr.107524.110 20644199 PMC2928508

[67] Jónsson H, Ginolhac A, Schubert M, Johnson PL, Orlando L. mapDamage2. 0: fast approximate Bayesian estimates of ancient DNA damage parameters. Bioinformatics. 2013;29(13):1682–1684. doi: 10.1093/bioinformatics/btt193 23613487 PMC3694634

[68] Okonechnikov K, Conesa A, García-Alcalde F. Qualimap 2: advanced multi-sample quality control for high-throughput sequencing data. Bioinformatics. 2016;32(2):292–294. doi: 10.1093/bioinformatics/btv566 26428292 PMC4708105

[69] Korneliussen TS, Albrechtsen A, Nielsen R. ANGSD: analysis of next generation sequencing data. BMC Bioinformatics. 2014;15(1):1–3. doi: 10.1186/s12859-014-0356-4 25420514 PMC4248462

[70] Billerman SM, Walsh J. Historical DNA as a tool to address key questions in avian biology and evolution: A review of methods, challenges, applications, and future directions. Molecular Ecology Resources. 2019;19(5):1115–1130. doi: 10.1111/1755-0998.13066 31336408

[71] Stamatakis A. RAxML version 8: a tool for phylogenetic analysis and post-analysis of large phylogenies. Bioinformatics. 2014;30(9):1312–1313. doi: 10.1093/bioinformatics/btu033 24451623 PMC3998144

[72] Meisner J, Albrechtsen A. Inferring population structure and admixture proportions in low-depth NGS data. Genetics. 2018;210(2):719–731. doi: 10.1534/genetics.118.301336 30131346 PMC6216594

[73] Pina-Martins F, Silva DN, Fino J, Paulo OS. Structure_threader: an improved method for automation and parallelization of programs structure, fastStructure and MavericK on multicore CPU systems. Molecular Ecology Resources. 2017;17(6):e268–e274. doi: 10.1111/1755-0998.12702 28776963

[74] Pritchard JK, Stephens M, Donnelly P. Inference of population structure using multilocus genotype data. Genetics. 2000;155(2):945–959. doi: 10.1093/genetics/155.2.945 10835412 PMC1461096

[75] Kopelman NM, Mayzel J, Jakobsson M, Rosenberg NA, Mayrose I. Clumpak: a program for identifying clustering modes and packaging population structure inferences across K. Molecular Ecology Resources. 2015;15(5):1179–1191. doi: 10.1111/1755-0998.12387 25684545 PMC4534335

[76] Soraggi S, Wiuf C, Albrechtsen A. Powerful inference with the D-statistic on low-coverage whole-genome data. G3: Genes, Genomes, Genetics. 2018;8(2):551–566. doi: 10.1534/g3.117.300192 29196497 PMC5919751

[77] Lawal RA, Martin SH, Vanmechelen K, Vereijken A, Silva P, Al-Atiyat RM, et al. The wild species genome ancestry of domestic chickens. BMC Biology. 2020;18(1):1–8. 32050971 10.1186/s12915-020-0738-1 PMC7014787

[78] Nishibori M, Shimogiri T, Hayashi T, Yasue H. Molecular evidence for hybridization of species in the genus *Gallus* except for *Gallus varius*. Animal Genetics. 2005;36(5):367–375. doi: 10.1111/j.1365-2052.2005.01318.x 16167978

[79] Emms DM, Kelly S. OrthoFinder: phylogenetic orthology inference for comparative genomics. Genome Biology. 2019;20(1):1–4. 31727128 10.1186/s13059-019-1832-y PMC6857279

[80] Ranwez V, Douzery EJ, Cambon C, Chantret N, Delsuc F. MACSE v2: toolkit for the alignment of coding sequences accounting for frameshifts and stop codons. Molecular Biology and Evolution. 2018;35(10):2582–2584. doi: 10.1093/molbev/msy159 30165589 PMC6188553

[81] Ranwez V, Harispe S, Delsuc F, Douzery EJ. MACSE: Multiple Alignment of Coding Sequences accounting for frameshifts and stop codons. PloS One. 2011;6(9):e22594. doi: 10.1371/journal.pone.0022594 21949676 PMC3174933

[82] Di Franco A, Poujol R, Baurain D, Philippe H. Evaluating the usefulness of alignment filtering methods to reduce the impact of errors on evolutionary inferences. BMC Evolutionary Biology. 2019;19(1):1–7. 30634908 10.1186/s12862-019-1350-2 PMC6330419

[83] McGowan PJK, Kirwan GM. Red Junglefowl (*Gallus gallus*), version 1.0. 2020 March 4 [cited 2022 April 29]. In Birds of the World [Internet]. New York: Cornell Lab of Ornithology. Available from: https://birdsoftheworld.org/bow/species/redjun/cur/introduction/.

