# Supplementary figures and images for: Historic samples reveal loss of wild genotype through domestic chicken introgression during the Anthropocene
Source: PLoS Genet. 2023 Jan 19;19(1):e1010551. doi: 10.1371/journal.pgen.1010551 (PMC9851510; doi:10.1371/journal.pgen.1010551)

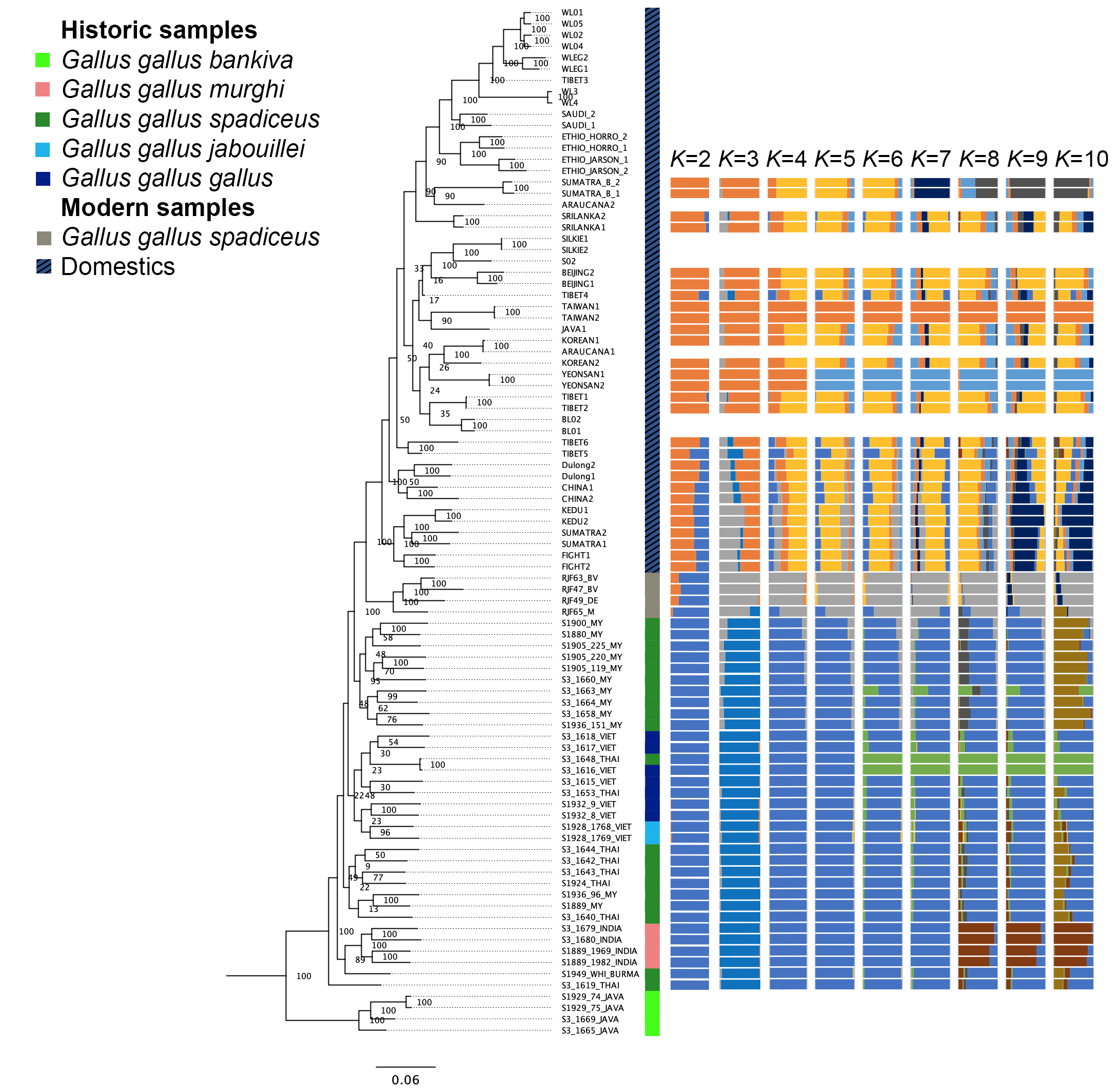

Supplement: S1 Fig — Phylogenetic relationships within Gallus gallus obtained from the concatenation of a subset of 1,172,919 single nucleotide polymorphisms harvested from whole genome resequenced samples using maximum-likelihood in RAxML (left). Bambusicola thoracicus was used as an outgroup (not shown). Numbers at nodes are bootstrap values. STRUCTURE plots for K = 2 to 10 based on 100,000 single nucleotide polymorphisms (right), run for a selection of domestic chickens and for all wild junglefowl samples except the divergent subspecies bankiva. For K = 2, the orange colour represents the domestic population contribution while blue represents the ‘wild-type’ contribution. Historic samples were collected between 1874 and 1939 and modern samples at the beginning of this century. (TIF) [file pgen.1010551.s001.tif]

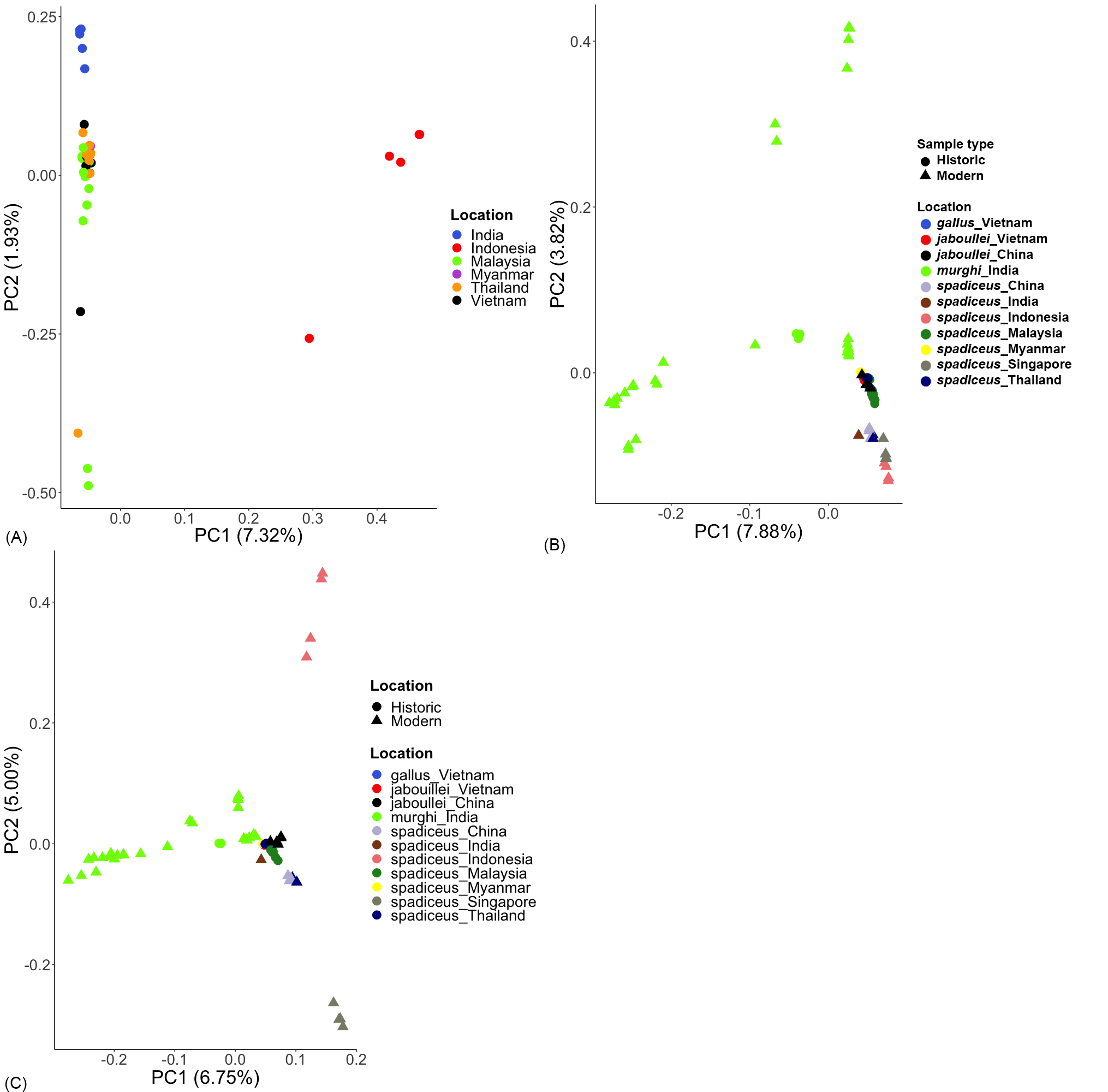

Supplement: S2 Fig — The percentage variation explained by each principal component (PC) is shown in brackets. (A) Historic wild junglefowl of all five subspecies. Historic red junglefowl of the Javan subspecies bankiva (labeled as ‘Indonesia’) were found to be widely divergent (right-hand side of plot) based on PC1. (B) Historic and modern samples of wild junglefowl of all non-bankiva subspecies. Modern and historic samples from many populations of red junglefowl (especially murghi from India) show substantial genetic separation. (C) Using the same dataset as in (B), the placement of historic samples was projected using the variation from the modern samples using smartpca. This analytical approach led to a correction of the position of low coverage modern Indian samples of subspecies murghi. Meanwhile, the placement of historic samples is similar to (B). Historic samples were collected between 1874 and 1939 and modern samples at the beginning of this century. (TIF) [file pgen.1010551.s002.tif]

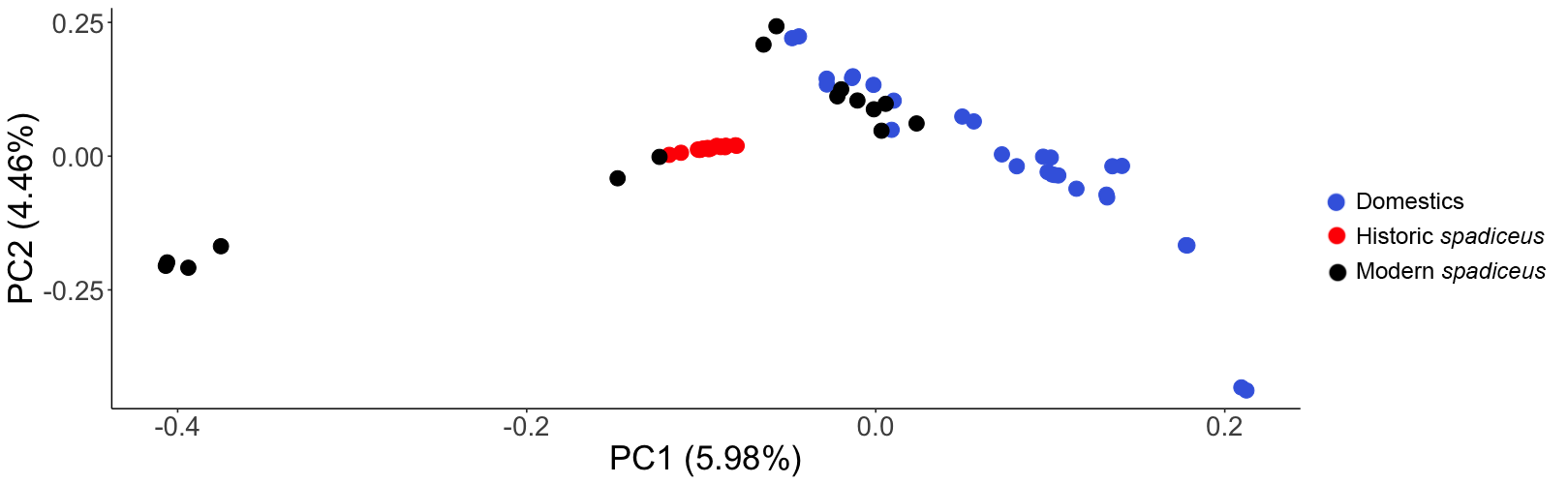

Supplement: S3 Fig — Placement of historic samples was projected using the variation from the modern samples. (TIF) [file pgen.1010551.s003.tif]

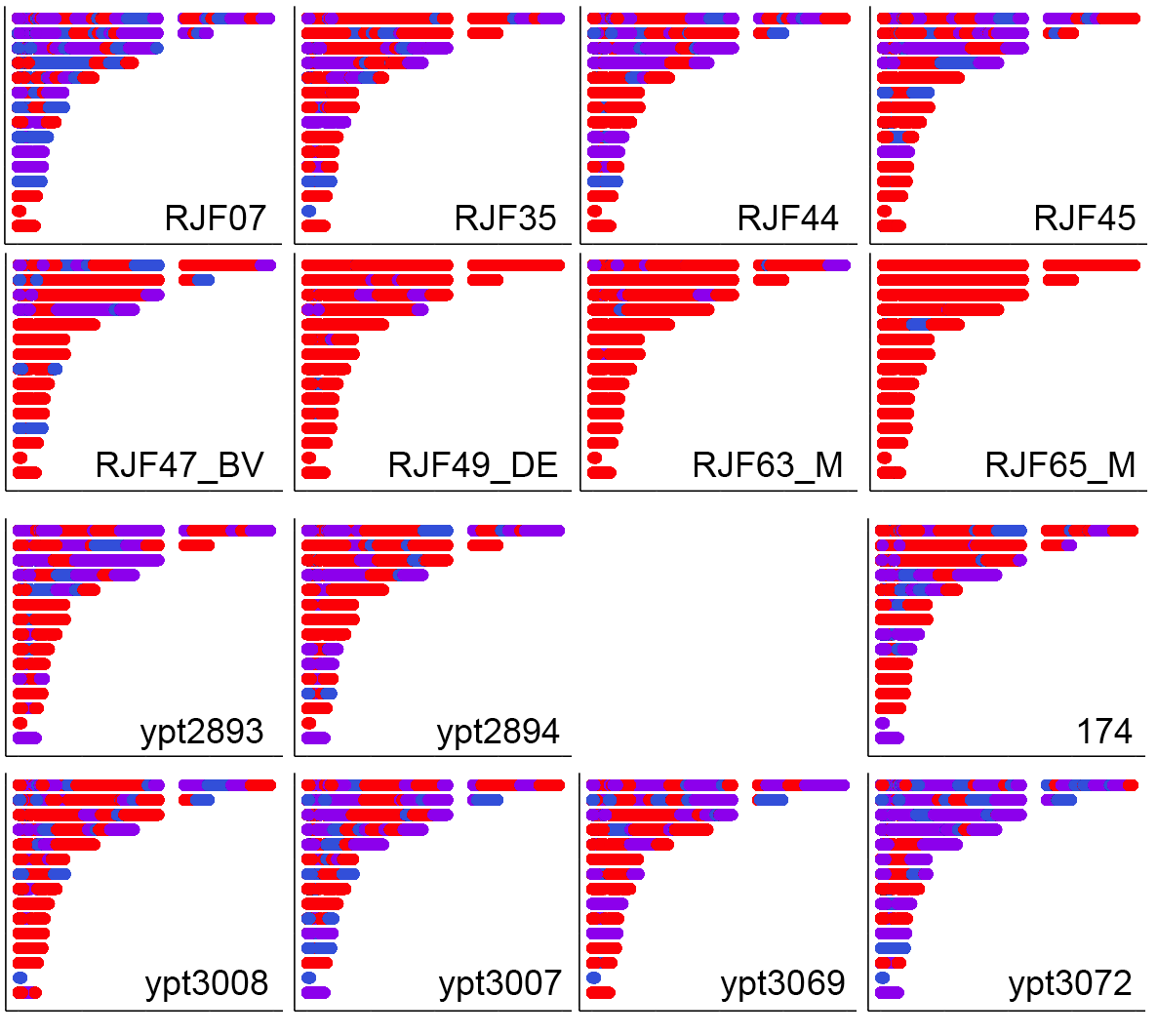

Supplement: S4 Fig — Only the top 15 chromosomes (Chr 1, 2, 3, 4, 5, 6, 7, 8, 9, 10, 12, 13, 14, 16, 20) with the most SNPs called are illustrated. Blue coloration refers to domestic ancestry, red coloration refers to wild ancestry, purple coloration refers to admixed ancestry. (TIF) [file pgen.1010551.s004.tif]

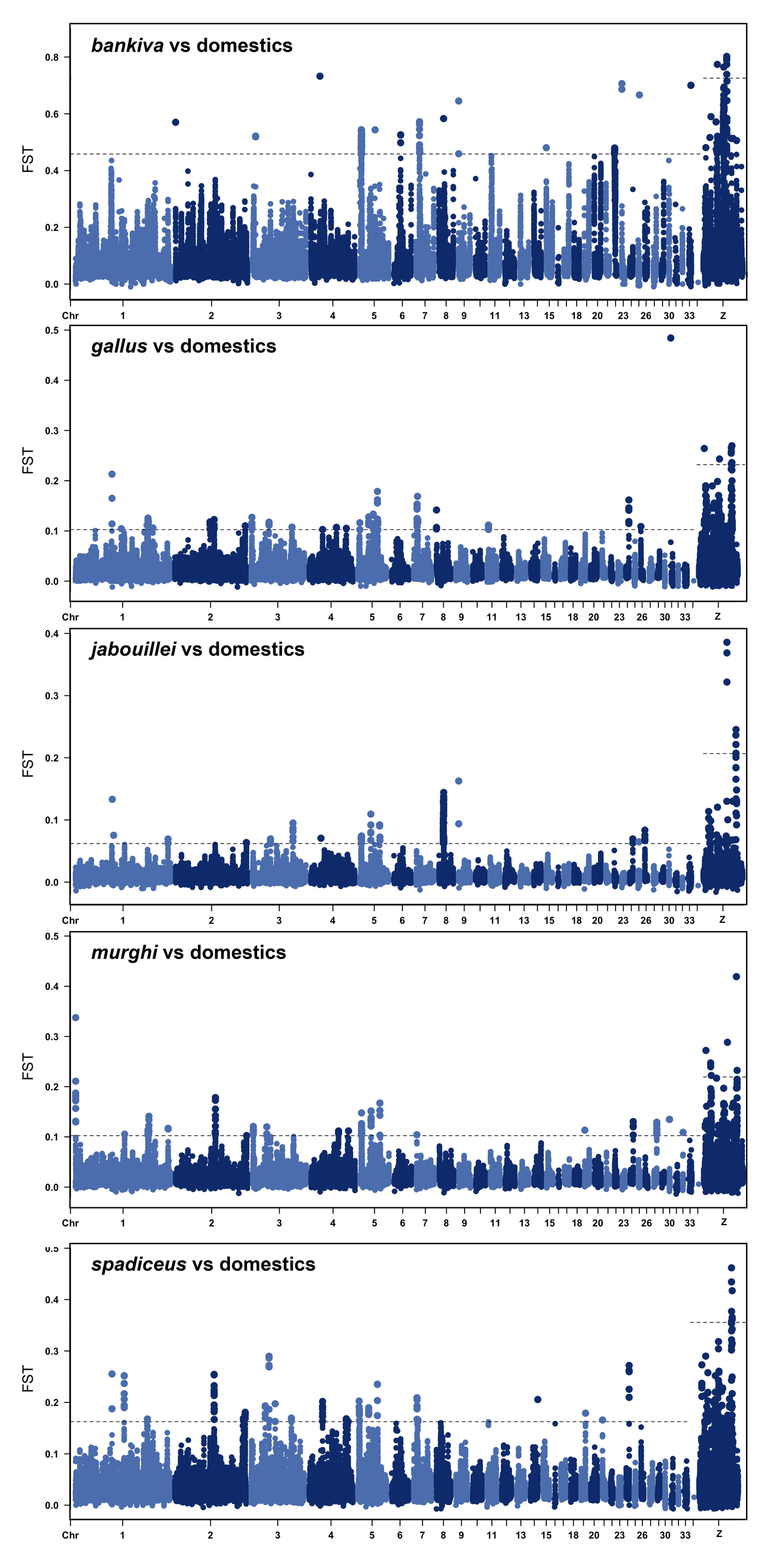

Supplement: S5 Fig — Alternating hues of blue denote different chromosomes and the horizontal black dotted lines denote the 99th percentile of FST of autosomes (bottom) and the Z chromosome (top) (TIF) [file pgen.1010551.s005.tif]

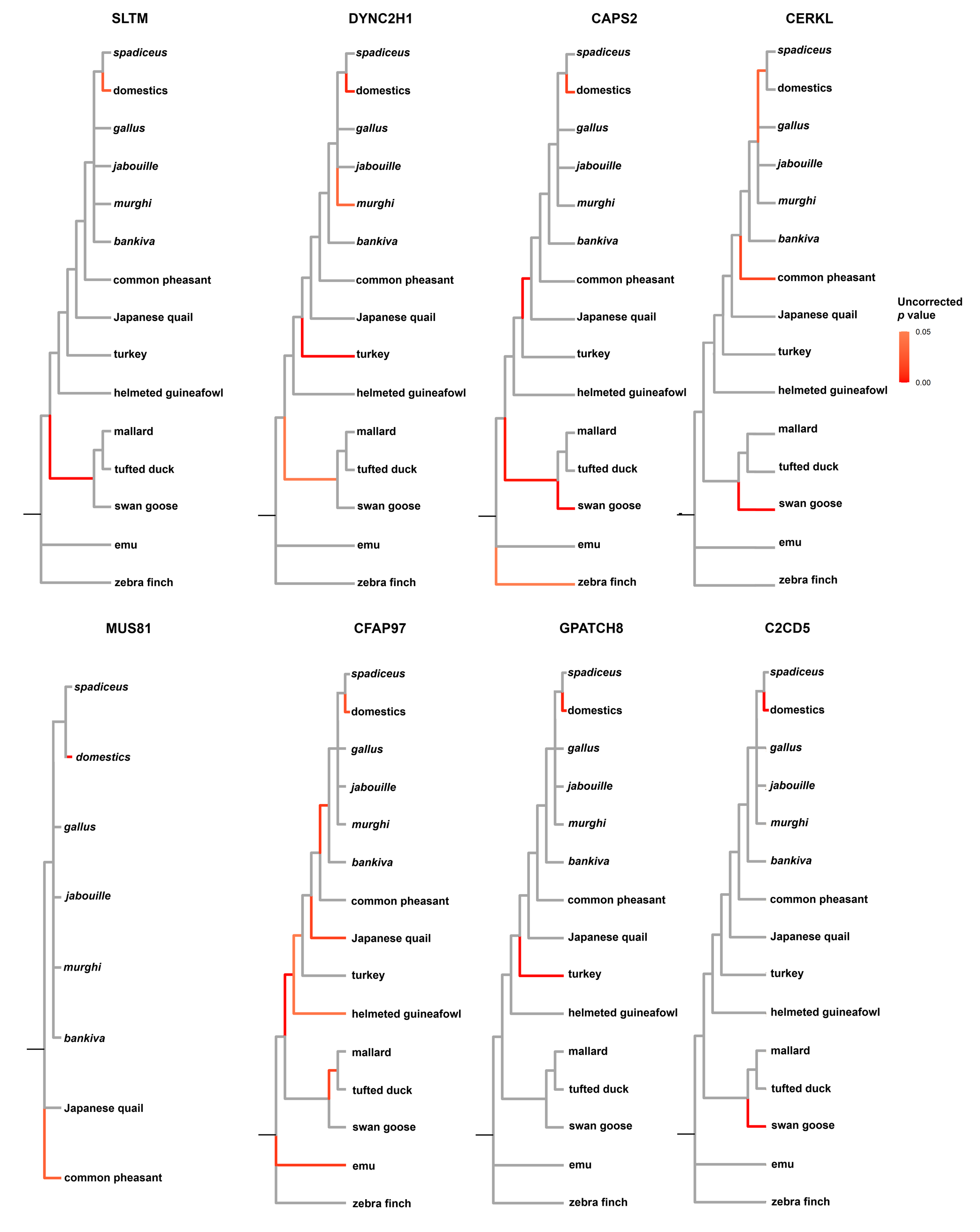

Supplement: S6 Fig — The input tree topology was given as the input gene tree topology of ((((((((((murghi, jabouille, gallus, (domestics, spadiceus)), bankiva), common pheasant), Japanese quail), turkey), helmeted guineafowl), (swan goose, (tufted duck, mallard))), zebra finch), emu)). Only two outgroups were present after quality filtering in MUS81. (TIF) [file pgen.1010551.s006.tif]

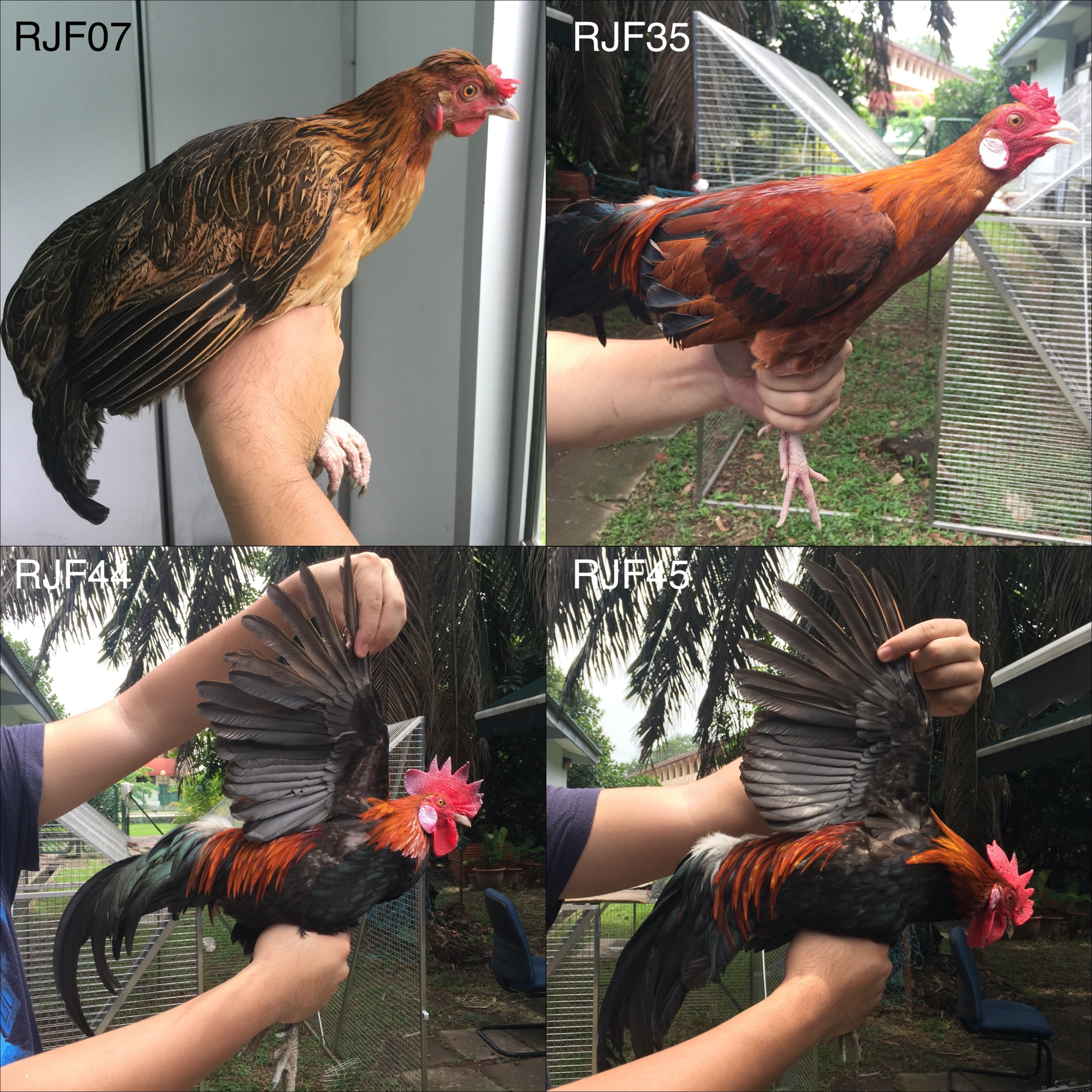

Supplement: S7 Fig — These individuals do not exhibit the wild phenotype for red junglefowl (see 1st table in Wu et al. 2020 [19]). [Photo courtesy of Gabriel Weijie Low]. (TIF) [file pgen.1010551.s007.tif]
